# Complete mitochondrial genome sequence and phylogenetic analysis of *Anisarchus medius* (Reinhardt, 1837)

**DOI:** 10.1080/23802359.2019.1689192

**Published:** 2019-11-13

**Authors:** Kun Liu, Heshan Lin, Jianjun Wang, Jun Sun

**Affiliations:** a College of Biotechnology, Tianjin University of Science and Technology, Tianjin, China;; b Laboratory of Marine Biology and Ecology, Third Institute of Oceanography, Ministry of Natural Resources, Xiamen, China

**Keywords:** *Anisarchus medius*, mitochondrial genome, Arctic fish, phylogenetic relationship

## Abstract

*Anisarchus medius* (Reinhardt, 1837) has been identified as a target species for investigating the effects of climate change on population patterns in coastal Arctic ecosystems. The complete mitochondrial genome of the *Anisarchus medius* was first sequenced in this study. The mitochondrial genome was sequenced with 16,524 bp in length, including 13 protein-coding genes, 22 transfer RNA genes, two ribosomal RNA genes, and a putative control region. All the protein-coding genes employed ATG as the start codon except COI using GTG. Three stop codons were observed in the protein-coding genes, including TAA, TAG and T. In addition, phylogenetic tree can provide further genetic analysis for this important species.

*Anisarchus medius* is a widely distributed Arctic fish species found on muddy bottoms at depths of 30–100 m, and generally preferring water below 0 °C (Whitehead et al. 1986). The distribution of the cold-water species is expected to recede northward as global ocean warming, restricting available habitat (Rose 2005). This large-scale range shift is expected to disrupt longstanding patterns of gene flow, species dispersal and effective population sizes (Swanburg et al. 2016). Whole mitochondrial genome studies can be used to assess the effect of climate change on the Arctic fish populations and serve as a baseline for future study (Swanburg et al. 2016).

The specimen (Tio-R08-22-2018) was collected from Chukchi Sea continental shelf (168.9°W, 71.2°N) during the 9th Chinese National Arctic Research Expedition in 2018 and deposited in the Marine Biological Sample Museum, Third Institute of Oceanography, Ministry of Natural Resources. In this study, the complete mitochondrial genome (mitogenome) of *Anisarchus medius* was sequenced and phylogenetic topology was reconstructed.

The complete mitogenome of *Anisarchus medius* is a circular molecule of 16,524 bp in length (GenBank number MN480302) and contains 13 protein-coding genes (PCGs), 22 tRNA genes, two rRNA genes (12S and 16S), and a putative control region. The gene order and organization were consistent with the found in other Stichaeidae fishes (Ayala et al., 2017; Turanov et al. 2019). Among these genes, one protein-coding gene ND6 and eight tRNAs genes (-Gln, -Ala, -Asn, -Cys, -Tyr, -Ser, -Glu, and -Pro) were located on the light strand (L-strand), the other genes were located on the heavy strand (H-strand). Four nucleoside base composition of the mitogenome was 27.4% for T, 28.1% for C, 27.0% for A, and 17.5% for G, respectively.

All 13 PCGs in this species employed ATG as the start codon, except for COI which used GTG. In addition, three stop codons were observed in these 13 PCGs, including TAG for ND3, T for three genes: COII, ND4 and Cyt *b*, TAA for other nine PCGs. Nine gaps and 11 overlaps existed in adjacent genes, such as ND1 (4 bp gaps), tRNA^Asp^ (10 bp gaps), tRNA^Glu^ (5 bp gaps), ATPase8 (10 bp overlaps), ND5 (4 bp overlaps) and ND4L (7 bp overlaps).

To explore the evolutionary position of *Anisarchus medius*, a neighbor-joining (NJ) tree was constructed based on 13 mitochondrial protein-coding genes of this species and three other Stichaeidae species using MEGA 6.06 (Tamura et al. 2013). The result showed that the taxonomic status of *Anisarchus medius* was closely related to three species of the family Stichaeidae, and *Anarhichas minor* in the family Anarhichadidae was used to root the trees (Figure 1). In conclusion, the complete mitochondrial genome sequence and phylogenetic relationship analysis of *Anisarchus medius* in this study can provide useful genetic information for future genetic variation identification and genetic diversity evaluation of this important species.

**Figure 1.**
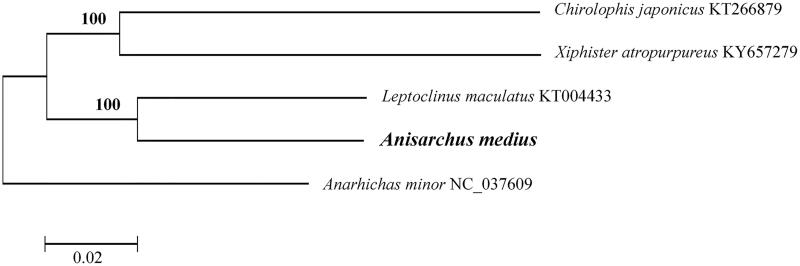
Phylogenetic relationships using NJ algorithm among 4 Stichaeidae species based on 13 mitochondrial protein-coding genes. *Anarhichas minor* in the family Anarhichadidae was used to root the trees.
